# Crystal structure and Hirshfeld surface analysis of (±)-*N*′-(2-hy­droxy-3-meth­oxy­benzyl­idene)-2-(4-iso­butyl­phen­yl)propionohydrazide

**DOI:** 10.1107/S2056989022007605

**Published:** 2022-07-29

**Authors:** Shaaban K. Mohamed, Joel T. Mague, Mehmet Akkurt, Laila H. Abdel-Rahman, Mohamed Abdel-Hameed, Mustafa R. Albayati, Elham A. Al-Taif

**Affiliations:** a Chemistry and Environmental Division, Manchester Metropolitan University, Manchester, M1 5GD, England; b Chemistry Department, Faculty of Science, Minia University, 61519 El-Minia, Egypt; c Department of Chemistry, Tulane University, New Orleans, LA 70118, USA; d Department of Physics, Faculty of Sciences, Erciyes University, 38039 Kayseri, Turkey; e Chemistry Department, Faculty of Science, Sohag University, 82534 Sohag, Egypt; f Department of Chemistry, College of Science, Kirkuk University, Kirkuk, Iraq; g Chemistry Department, Faculty of Science, Sana’a University, Sana’a, Yemen; University of Neuchâtel, Switzerland

**Keywords:** crystal structure, hydrogen bond, Schiff base, ibuprofen, Hirshfeld surface analysis, hydrazide

## Abstract

The title mol­ecule, C_21_H_26_N_2_O_3_, adopts a V-shaped conformation and is chiral at the C atom with methyl group attached at the common cut of the edges of the V-conformation and crystallizes as a racemate. It also contains an intra­molecular O—H⋯N hydrogen bond. In the crystal, N—H⋯O hydrogen bonds form chains of mol­ecules extending along the *c*-axis direction, together with normal van der Waals contacts. The roles of the various inter­molecular inter­actions were clarified by Hirshfeld surface analysis, which reveals that the most important contributions to the crystal packing are from H⋯H (62.6%), C⋯H/H⋯C (15.8%) and O⋯H/H⋯O (15.3%) contacts.

## Abstract 2

The title mol­ecule adopts a V-shaped conformation and contains an intra­molecular O—H⋯N hydrogen bond. In the crystal, N—H⋯O hydrogen bonds form chains of mol­ecules extending along the *c*-axis direction, which pack with normal van der Waals contacts.

## 1. Chemical context

Non-steroidal anti-inflammatory drugs (NSAIDs) are commonly used as analgesics and anti­pyretics to manage pain and inflammation in people with chronic pain, osteoarthritis, rheumatoid arthritis, postoperative surgical conditions, and menstrual cramps (Manzano *et al.*, 2018 ▸; Gupta & Bah, 2016 ▸; Budoff, 1979 ▸). Azo-methine structure-based ibuprofen core compounds in particular have been used as anti-viral and anti-bacterial agents (El Bakri *et al.*, 2022 ▸). Based on such significant activity, we herein report the crystal structure of a member of this family, namely (±)-*N*′-(2-hy­droxy-3-meth­oxy­benzyl­idene)-2-(4-iso­butyl­phen­yl)propionohydrazide. 
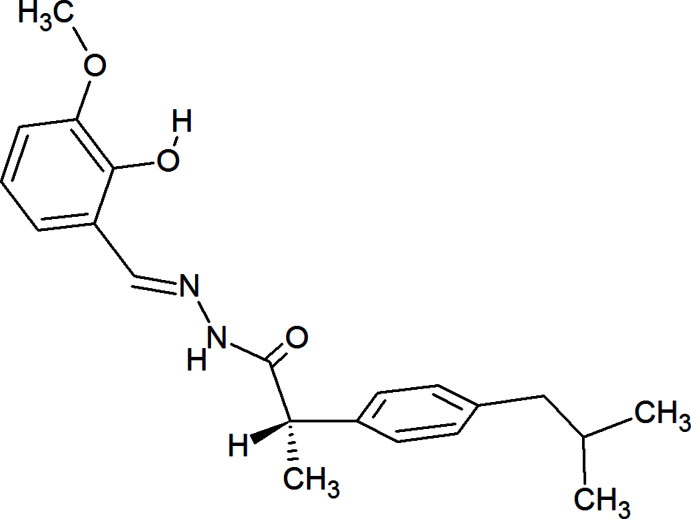


## 2. Structural commentary

**Figure 1:**
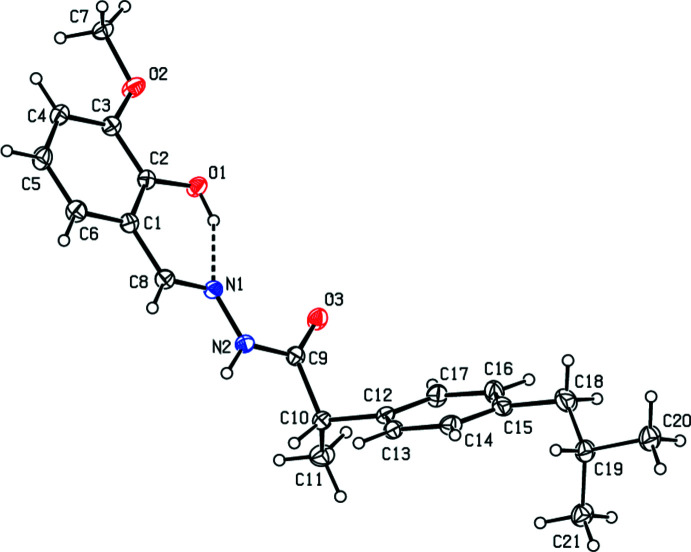
The title mol­ecule with labeling scheme and 30% probability level ellipsoids. The intra­molecular O—H⋯N hydrogen bond is depicted by a dashed line. Only the major component of the disorder is shown.

In the solid state, the mol­ecule adopts a wide, V-shaped conformation (Fig. 1 ▸) with a dihedral angle of 1.08 (11)° between the mean plane of the C1–C6 ring and the chain defined by C8, C9, N1 and N2. This is likely due to the intra­molecular O1—H1⋯N1 hydrogen bond (Table 1 ▸ and Fig. 1 ▸). The dihedral angle between the latter chain and the mean plane of the C12–C17 ring is 59.34 (6)°. There is one stereogenic center in the racemic title compound and the chirality of the C10 atom is *S* in the chosen asymmetric unit. All bond distances and angles appear as expected.

## 3. Supra­molecular features and Hirshfeld surface analysis

**Figure 2:**
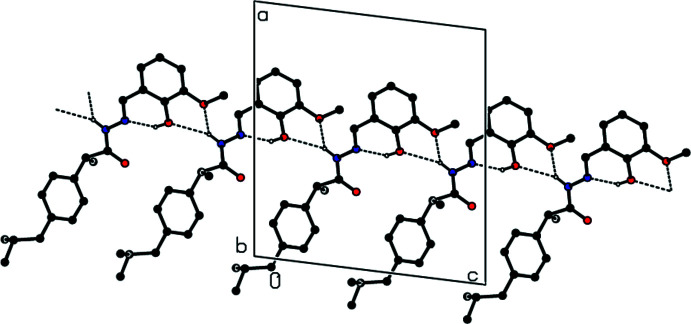
A portion of one chain viewed along the *b*-axis with the O—H⋯N and N—H⋯O hydrogen bonds depicted by dashed lines and non-inter­acting hydrogen atoms omitted for clarity.

In the crystal, N2—H2⋯O2 and weaker N2—H2⋯O1 hydrogen bonds (Table 1 ▸) form chains of mol­ecules extending along the *c*-axis direction (Fig. 2 ▸). The mol­ecular packing is provided by normal van der Waals inter­actions between chains.

**Figure 3:**
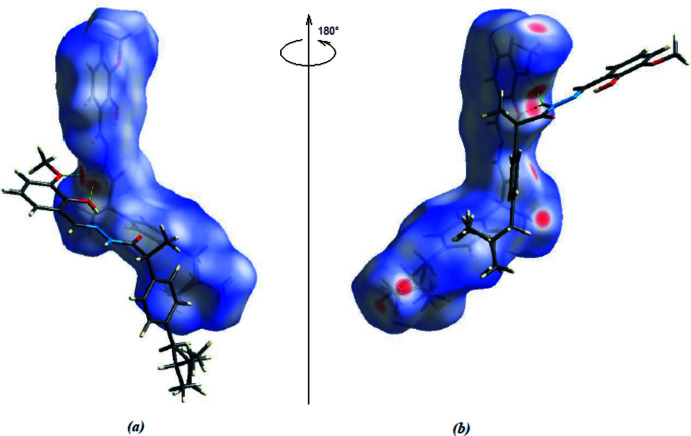
(*a*) Front view and (*b*) back view of the three-dimensional Hirshfeld surface of the title compound plotted over *d*_norm_ in the range −0.3801 to +1.4738 a.u. The red, white and blue regions visible on the *d*_norm_ surfaces indicate contacts with distances shorter, longer and equal to the van der Waals separations, respectively. The red spots highlight the inter­atomic contacts, including the O—H⋯N and N—H⋯O hydrogen bonds.

Hirshfeld surfaces and their related two-dimensional fingerprint plots were generated using *CrystalExplorer17.5* (Turner *et al.*, 2017 ▸) to visually represent the inter­molecular inter­actions in the crystal structure of the title compound. The Hirshfeld surface plotted over *d*_norm_ in the range −0.3801 to +1.4738 a.u. is shown in Fig. 3 ▸. The inter­actions shown in Tables 1 ▸ and 2 ▸ are important in the mol­ecular packing of the title compound.

**Figure 4:**
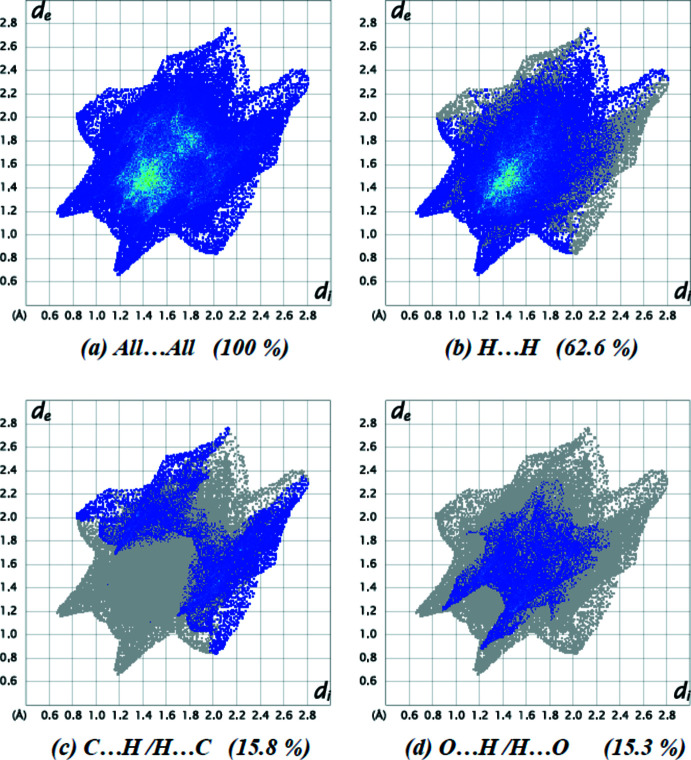
Two-dimensional fingerprint plots for the title compound, showing (*a*) all inter­actions, and delineated into (*b*) H⋯H, (*c*) C⋯H/H⋯C and (*d*) O⋯H/H⋯O inter­actions. The *d*_i_ and *d*_e_ values are the closest inter­nal and external distances (in Å) from given points on the Hirshfeld surface.

The overall two-dimensional fingerprint plot is illustrated in Fig. 4 ▸ *a*, and those delineated into the major contacts: H⋯H (62.6%; Fig. 4 ▸ *b*), C⋯H/H⋯C (15.8%; Fig. 4 ▸ *c*), O⋯H/H⋯O and (15.3%; Fig. 4 ▸ *d*). The other contacts are negligible with individual contributions of less than 2.2% [N⋯H/H⋯N (2.2%), N⋯C/C⋯N (2.1%), C⋯C (1.3%) and N⋯C/C⋯N (0.7%)].

## 4. Database survey

Six related compounds were found in a search of the Cambridge Structural Database (CSD, version 5.42, update of September 2021; Groom *et al.*, 2016 ▸), *viz. N*′-benzyl­idene-2-({5-[(4-chloro­phen­oxy)meth­yl]-4-phenyl-4*H*-1,2,4-triazol-3-yl}sulfan­yl)acetohydrazide hemihydrate [CSD refcode ULARIK (**I**); Mague *et al.*, 2016 ▸], *N*′-[(3-cyano­phen­yl)meth­yl­idene]-*N*-methyl-2-(thio­phen-2-yl)acetohydrazide [ECO­WEB (**II**); Cardoso *et al.*, 2017 ▸], *N*′-[(4-meth­oxy­phen­yl)methyl­idene]-*N*-methyl-2-(thio­phen-2-yl)acetohydrazide [ECO­WIF (**III**); Cardoso *et al.*, 2017 ▸], *N*′-[(1*Z*)-1-(3-methyl-5-oxo-1-phenyl-1,5-di­hydro-4*H*-pyrazol-4-yl­idene)eth­yl]-2-[(4-methyl­phen­yl)sulfan­yl]acetohydrazide [GEMQIB (**IV**); Mohamed *et al.*, 2017 ▸], (*E*)-*N*′-(4-fluoro­benzyl­idene)-2-(3-methyl­phen­yl)acetohydrazide [MEWMUY (**V**); Praveen *et al.*, 2013 ▸] and *N*′-[4-(di­methyl­amino)­benzyl­idene]-2-(4-methyl­phen­oxy)aceto­hydrazide [ZIYSOR (**VI**); Usha *et al.*, 2014 ▸].

In (**I**), three independent mol­ecules in the asymmetric unit and two water mol­ecules of crystallization are observed. The three unique organic mol­ecules differ in the conformations of the substituents on the pyrazole ring. In the crystal, extensive O—H⋯O, O—H⋯N, N—H⋯O and C—H⋯O hydrogen bonding generates a three-dimensional network and C—H⋯π inter­actions are also observed. Compounds (**II**) and (**III**) crystallize with two mol­ecules in the asymmetric unit, with generally similar conformations that approximate to L-shapes. The packing for (**II**) features short C—H⋯O inter­actions arising from the C—H adjacent to the cyanide group and C—H⋯N_c_ (c = cyanide) links arising from the methine groups to generate [110] double chains. Weak C—H⋯π inter­actions inter­link the chains into a three-dimensional network. The packing for (**III**) features numerous C—H⋯O and C—H⋯π inter­actions arising from different donor groups to generate a three-dimensional network. In (**IV**), the mol­ecular conformation is influenced by intra­molecular N—H⋯O and C—H⋯O hydrogen bonds. In the crystal, N—H⋯O hydrogen bonds plus C—H⋯π and π–π stacking inter­actions lead to the formation of chains extending in the *a*-axis direction. The chains are linked by complementary pairs of C—H⋯π inter­actions. Compound (**V**) has four independent mol­ecules in the asymmetric unit. In the crystal, N—H—O hydrogen bonds involving the hydrazide and acetyl groups, which form 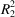 (18) ring motifs, link the mol­ecules into dimers, which form columns along the [010] plane. In the crystal of (**VI**), the mol­ecules are linked by C—H⋯O and N—H⋯O hydrogen bonds, as well as weak C—H⋯π contacts, forming a three-dimensional supra­molecular architecture.

## 5. Synthesis and crystallization

The title compound was synthesized by mixing 1.101g (5 mmol) of ibuprofen hydrazide in 15 mL of chloro­form with 0.76 g (5 mmol) of 2-hy­droxy-3-meth­oxy­benzaldehyde in 15 mL of methanol. A few drops of acetic acid were added to the reaction mixture as catalyst and the mixture was refluxed at 333 K for 1 h. The reaction progress was monitored by TLC until completion. The crude product as a pale-yellow precipitate was filtered off, washed, recrystallized from ethanol and dried under vacuum over anhydrous CaCl_2_ under vacuum. M.p. 444.15 K; 87% yield.

The product was characterized by different spectroscopic analyses. Empirical formula, C_21_H_26_N_2_O_3_ (354.33 g mol^−1^); IR (cm^−1^); 3280 (NH), 1704 (C=O), 1612 (C=N), and 1248 (C—O). ^1^H NMR (400 MHz, CDCl_3_) ppm δ = 0.83–0.86 (*d*, *J* = 6.6 Hz, 6H), 1.37–1.43 (*d*, *J* = 7.0 Hz, 3H), 1.45–1.84 (*m*, 1H), 2.37–2.52 (*d*, *J* = 7.1 Hz, 2H), 3.65–3.70 (*q*, *J* = 7.0 Hz, 3H), 3.80–3.82 (*s*, 3H), 6.81–7.30 (*m*, 7H), 8.41 (*s*, 1H), 10.82 (*s*, 1H), 11.73 (*s*, 1H). ^13^C NMR (75 MHz, CDCl_3_) δ = 18.88, 19.10, 22.64, 30.10, 39.55, 39.97, 40.38, 44.70, 56.28, 113.19, 114.12, 118.34, 119.68, 121.12, 127.68, 129.46, 139.72, 140.14, 146.31, 148.34, 170.12.

## 6. Refinement

Crystal data, data collection and structure refinement details are summarized in Table 3 ▸. H atoms attached to carbon were placed in calculated positions (C—H = 0.95–1.00 Å) and were included as riding contributions with isotropic displacement parameters 1.2–1.5 times those of the attached atoms. Those attached to nitro­gen and to oxygen were placed in locations derived from a difference map and refined freely with DFIX 0.91 0.01 and DFIX 0.84 0.01 instructions, respectively. The atoms of the propane group are disordered over two sets of sites with an occupancy ratio of 0.929 (3):0.071 (3).

## Supplementary Material

Crystal structure: contains datablock(s) I. DOI: 10.1107/S2056989022007605/tx2052sup1.cif

Structure factors: contains datablock(s) I. DOI: 10.1107/S2056989022007605/tx2052Isup2.hkl

Supporting information file. DOI: 10.1107/S2056989022007605/tx2052Isup3.cml

CCDC reference: 2192678

Additional supporting information:  crystallographic information; 3D view; checkCIF report

## Figures and Tables

**Table 1:** Hydrogen-bond geometry (Å, °)

*D*—H⋯*A*	*D*—H	H⋯*A*	*D*⋯*A*	*D*—H⋯*A*
O1—H*O*1⋯N1	0.85 (1)	1.83 (1)	2.5914 (13)	149 (2)
N2—H*N*2⋯O1^i^	0.90 (1)	2.40 (1)	3.2470 (14)	157 (1)
N2—H*N*2⋯O2^i^	0.90 (1)	2.18 (1)	2.8745 (14)	133 (1)

Symmetry code: (i) 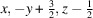 .

**Table 2:** Summary of short inter­atomic contacts (Å) in the title compound

Contact	Distance	Symmetry operation
H*O*1⋯H7*C*	2.49	1 − *x*, − 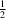 + *y*, 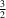 − *z*
O2⋯H*N*2	2.18	*x*, 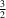 − *y*, 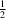 + *z*
H13⋯H7*C*	2.47	1 − *x*, 2 − *y*, 1 − *z*
H11*B*⋯C2	2.95	1 − *x*, 1 − *y*, 1 − *z*
C6⋯H19*A*	2.90	1 + *x*, 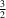 − *y*, 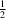 + *z*
H6⋯C13	2.98	1 − *x*, 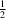 + *y*, 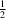 − *z*
H7*A*⋯H20*D*	2.26	1 + *x*, *y*, 1 + *z*
H11*A*⋯H20*F*	2.05	−*x*, − 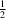 + *y*, 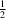 − *z*
H20*C*⋯H14	2.58	-*x*, 2 − *y*, −*z*
H21*D*⋯H21*A*	2.02	-*x*, 1 − *y*, −*z*

**Table 3:** Experimental details

Crystal data	
Chemical formula	C_21_H_26_N_2_O_3_
*M*_r_	354.44
Crystal system, space group	Monoclinic, *P*2_1_/*c*
Temperature (K)	125
*a*, *b*, *c* (Å)	14.5241 (7), 10.0718 (5), 13.2710 (7)
β (°)	97.042 (2)
*V* (Å^3^)	1926.69 (17)
*Z*	4
Radiation type	Cu *K*α
μ (mm^−1^)	0.66
Crystal size (mm)	0.20 × 0.20 × 0.03

Data collection	
Diffractometer	Bruker D8 VENTURE PHOTON 3 CPAD
Absorption correction	Multi-scan (*SADABS*; Krause *et al.*, 2015 ▸)
*T*_min_, *T*_max_	0.90, 0.98
No. of measured, independent and observed [*I* > 2σ(*I*)] reflections	38584, 3758, 3400
*R*_int_	0.035
(sin θ/λ)_max_ (Å^−1^)	0.618

Refinement	
*R*[*F* ^2^ > 2σ(*F* ^2^)], *wR*(*F* ^2^), *S*	0.041, 0.109, 1.06
No. of reflections	3758
No. of parameters	259
No. of restraints	8
H-atom treatment	H atoms treated by a mixture of independent and constrained refinement
Δρ_max_, Δρ_min_ (e Å^−3^)	0.40, −0.21

Computer programs: *APEX3* and *SAINT* (Bruker, 2016 ▸), *SHELXT* (Sheldrick, 2015*a* ▸), *SHELXL2018/1* (Sheldrick, 2015*b* ▸), *ORTEP-3 for Windows* (Farrugia, 2012 ▸) and *PLATON* (Spek, 2020 ▸).

## supplementary crystallographic information

### Crystal data

**Table:**

C_21_H_26_N_2_O_3_	*F*(000) = 760
*M**_r_* = 354.44	*D*_x_ = 1.222 Mg m^−^^3^
Monoclinic, *P*2_1_/*c*	Cu *K*α radiation, λ = 1.54178 Å
*a* = 14.5241 (7) Å	Cell parameters from 9778 reflections
*b* = 10.0718 (5) Å	θ = 5.5–72.4°
*c* = 13.2710 (7) Å	µ = 0.66 mm^−^^1^
β = 97.042 (2)°	*T* = 125 K
*V* = 1926.69 (17) Å^3^	Plate, colourless
*Z* = 4	0.20 × 0.20 × 0.03 mm

### Data collection

**Table:**

Bruker D8 VENTURE PHOTON 3 CPAD diffractometer	3758 independent reflections
Radiation source: INCOATEC IµS micro—-focus source	3400 reflections with *I* > 2σ(*I*)
Mirror monochromator	*R*_int_ = 0.035
Detector resolution: 7.3910 pixels mm^-1^	θ_max_ = 72.4°, θ_min_ = 5.5°
φ and ω scans	*h* = −17→17
Absorption correction: multi-scan (*SADABS*; Krause *et al.*, 2015)	*k* = −12→12
*T*_min_ = 0.90, *T*_max_ = 0.98	*l* = −14→16
38584 measured reflections	

### Refinement

**Table:**

Refinement on *F*^2^	8 restraints
Least-squares matrix: full	Hydrogen site location: mixed
*R*[*F*^2^ > 2σ(*F*^2^)] = 0.041	H atoms treated by a mixture of independent and constrained refinement
*wR*(*F*^2^) = 0.109	*w* = 1/[σ^2^(*F*_o_^2^) + (0.0517*P*)^2^ + 0.694*P*] where *P* = (*F*_o_^2^ + 2*F*_c_^2^)/3
*S* = 1.06	(Δ/σ)_max_ < 0.001
3758 reflections	Δρ_max_ = 0.40 e Å^−^^3^
259 parameters	Δρ_min_ = −0.21 e Å^−^^3^

### Special details

**Table:**

Experimental. The diffraction data were obtained from 15 sets of frames, each of width 0.5° in ω or φ, collected with scan parameters determined by the "strategy" routine in *APEX4*. The scan time was 10 sec/frame.
Geometry. All esds (except the esd in the dihedral angle between two l.s. planes) are estimated using the full covariance matrix. The cell esds are taken into account individually in the estimation of esds in distances, angles and torsion angles; correlations between esds in cell parameters are only used when they are defined by crystal symmetry. An approximate (isotropic) treatment of cell esds is used for estimating esds involving l.s. planes.

### Fractional atomic coordinates and isotropic or equivalent isotropic displacement parameters (Å^2^)

**Table:**

	*x*	*y*	*z*	*U*_iso_*/*U*_eq_	Occ. (<1)
O1	0.48117 (6)	0.83125 (10)	0.62950 (7)	0.0328 (2)	
HO1	0.4586 (13)	0.7973 (19)	0.5735 (10)	0.061 (6)*	
O2	0.57158 (7)	0.95029 (10)	0.78175 (7)	0.0378 (2)	
O3	0.30167 (6)	0.64479 (10)	0.44100 (7)	0.0353 (2)	
N1	0.46923 (7)	0.75956 (10)	0.44081 (8)	0.0276 (2)	
N2	0.42448 (7)	0.69530 (11)	0.35782 (8)	0.0291 (2)	
HN2	0.4561 (10)	0.6800 (17)	0.3046 (10)	0.041 (4)*	
C1	0.59869 (9)	0.88043 (12)	0.51948 (9)	0.0272 (3)	
C2	0.56349 (8)	0.88651 (12)	0.61296 (9)	0.0259 (3)	
C3	0.61408 (9)	0.95151 (12)	0.69520 (9)	0.0276 (3)	
C4	0.69830 (9)	1.01023 (13)	0.68500 (10)	0.0310 (3)	
H4	0.732144	1.054394	0.741015	0.037*	
C5	0.73341 (9)	1.00426 (15)	0.59170 (11)	0.0364 (3)	
H5	0.791294	1.044688	0.584278	0.044*	
C6	0.68465 (9)	0.94019 (14)	0.51054 (10)	0.0341 (3)	
H6	0.709452	0.936307	0.447606	0.041*	
C7	0.61541 (9)	1.02016 (14)	0.86811 (10)	0.0343 (3)	
H7A	0.675774	0.979590	0.890582	0.051*	
H7B	0.576255	1.015696	0.923125	0.051*	
H7C	0.624321	1.113185	0.849937	0.051*	
C8	0.54787 (9)	0.81416 (13)	0.43243 (9)	0.0289 (3)	
H8	0.572764	0.811269	0.369529	0.035*	
C9	0.34188 (9)	0.63537 (13)	0.36611 (9)	0.0277 (3)	
C10	0.30483 (9)	0.55529 (13)	0.27208 (9)	0.0293 (3)	
H10	0.352167	0.557419	0.223374	0.035*	
C11	0.29224 (12)	0.41096 (14)	0.30427 (12)	0.0427 (4)	
H11A	0.250084	0.407883	0.356498	0.064*	
H11B	0.352538	0.373853	0.331631	0.064*	
H11C	0.266064	0.358829	0.245264	0.064*	
C12	0.21646 (8)	0.61920 (12)	0.22138 (9)	0.0268 (3)	
C13	0.21756 (9)	0.68811 (13)	0.13073 (9)	0.0287 (3)	
H13	0.273825	0.694597	0.101326	0.034*	
C14	0.13798 (10)	0.74751 (13)	0.08254 (10)	0.0340 (3)	
H14	0.140562	0.794801	0.021011	0.041*	
C15	0.05427 (10)	0.73865 (15)	0.12330 (10)	0.0377 (3)	
C16	0.05360 (10)	0.66893 (17)	0.21379 (11)	0.0419 (4)	
H16	−0.002864	0.660731	0.242608	0.050*	
C17	0.13317 (10)	0.61141 (16)	0.26259 (10)	0.0364 (3)	
H17	0.130938	0.566028	0.324999	0.044*	
C18	−0.03439 (12)	0.80231 (19)	0.07374 (12)	0.0507 (4)	
H18A	−0.087137	0.761937	0.103331	0.061*	
H18B	−0.032812	0.897690	0.091900	0.061*	
C19	−0.05346 (11)	0.79118 (15)	−0.04059 (12)	0.0363 (4)	0.929 (3)
H19	−0.002595	0.838797	−0.070091	0.044*	0.929 (3)
C20	−0.14474 (15)	0.8607 (2)	−0.07853 (17)	0.0478 (5)	0.929 (3)
H20A	−0.196295	0.813447	−0.053316	0.072*	0.929 (3)
H20B	−0.153574	0.860749	−0.152939	0.072*	0.929 (3)
H20C	−0.142860	0.952371	−0.053670	0.072*	0.929 (3)
C21	−0.05494 (17)	0.6501 (2)	−0.07825 (16)	0.0410 (5)	0.929 (3)
H21A	0.004431	0.607334	−0.054798	0.062*	0.929 (3)
H21B	−0.064915	0.649641	−0.152632	0.062*	0.929 (3)
H21C	−0.105315	0.601541	−0.051829	0.062*	0.929 (3)
C19A	−0.1011 (12)	0.7500 (17)	−0.0070 (13)	0.0363 (4)	0.071 (3)
H19A	−0.140915	0.702544	0.037706	0.044*	0.071 (3)
C20A	−0.172 (2)	0.852 (3)	−0.047 (3)	0.0478 (5)	0.071 (3)
H20D	−0.224290	0.807511	−0.087183	0.072*	0.071 (3)
H20E	−0.143533	0.914905	−0.090648	0.072*	0.071 (3)
H20F	−0.193809	0.899507	0.009485	0.072*	0.071 (3)
C21A	−0.064 (3)	0.629 (3)	−0.054 (3)	0.0410 (5)	0.071 (3)
H21D	−0.072593	0.551247	−0.010781	0.062*	0.071 (3)
H21E	0.001967	0.640502	−0.059041	0.062*	0.071 (3)
H21F	−0.097861	0.614437	−0.121356	0.062*	0.071 (3)

### Atomic displacement parameters (Å^2^)

**Table:**

	*U*^11^	*U*^22^	*U*^33^	*U*^12^	*U*^13^	*U*^23^
O1	0.0293 (5)	0.0422 (5)	0.0276 (5)	−0.0105 (4)	0.0059 (4)	−0.0063 (4)
O2	0.0384 (5)	0.0494 (6)	0.0260 (5)	−0.0148 (4)	0.0054 (4)	−0.0083 (4)
O3	0.0315 (5)	0.0495 (6)	0.0245 (5)	−0.0024 (4)	0.0017 (4)	−0.0011 (4)
N1	0.0287 (5)	0.0295 (5)	0.0238 (5)	0.0024 (4)	−0.0005 (4)	−0.0023 (4)
N2	0.0290 (5)	0.0352 (6)	0.0225 (5)	0.0011 (4)	0.0012 (4)	−0.0047 (4)
C1	0.0279 (6)	0.0265 (6)	0.0270 (6)	0.0017 (5)	0.0025 (5)	0.0029 (5)
C2	0.0246 (6)	0.0252 (6)	0.0278 (6)	−0.0002 (5)	0.0025 (5)	0.0027 (5)
C3	0.0291 (6)	0.0278 (6)	0.0257 (6)	0.0003 (5)	0.0024 (5)	0.0020 (5)
C4	0.0291 (6)	0.0307 (6)	0.0315 (6)	−0.0022 (5)	−0.0028 (5)	0.0013 (5)
C5	0.0287 (6)	0.0412 (7)	0.0395 (7)	−0.0078 (6)	0.0052 (5)	0.0017 (6)
C6	0.0330 (7)	0.0397 (7)	0.0306 (7)	−0.0043 (6)	0.0080 (5)	0.0022 (5)
C7	0.0349 (7)	0.0394 (7)	0.0275 (6)	−0.0025 (6)	−0.0008 (5)	−0.0065 (5)
C8	0.0308 (6)	0.0306 (6)	0.0255 (6)	0.0013 (5)	0.0039 (5)	0.0016 (5)
C9	0.0286 (6)	0.0309 (6)	0.0226 (6)	0.0050 (5)	−0.0005 (5)	0.0015 (5)
C10	0.0290 (6)	0.0327 (7)	0.0255 (6)	0.0021 (5)	0.0000 (5)	−0.0024 (5)
C11	0.0522 (9)	0.0326 (7)	0.0414 (8)	0.0032 (6)	−0.0021 (7)	−0.0002 (6)
C12	0.0277 (6)	0.0281 (6)	0.0235 (6)	−0.0009 (5)	−0.0009 (5)	−0.0044 (5)
C13	0.0287 (6)	0.0303 (6)	0.0270 (6)	−0.0027 (5)	0.0029 (5)	−0.0034 (5)
C14	0.0414 (7)	0.0330 (7)	0.0265 (6)	0.0032 (6)	−0.0002 (5)	0.0003 (5)
C15	0.0359 (7)	0.0467 (8)	0.0289 (7)	0.0122 (6)	−0.0025 (5)	−0.0095 (6)
C16	0.0296 (7)	0.0668 (10)	0.0297 (7)	0.0047 (6)	0.0059 (5)	−0.0063 (6)
C17	0.0333 (7)	0.0531 (8)	0.0230 (6)	−0.0013 (6)	0.0036 (5)	0.0009 (6)
C18	0.0439 (9)	0.0652 (11)	0.0409 (8)	0.0229 (8)	−0.0032 (7)	−0.0096 (7)
C19	0.0341 (8)	0.0353 (8)	0.0376 (8)	0.0053 (6)	−0.0039 (6)	0.0017 (6)
C20	0.0424 (11)	0.0441 (9)	0.0527 (12)	0.0110 (8)	−0.0112 (8)	0.0018 (9)
C21	0.0414 (10)	0.0356 (10)	0.0431 (13)	0.0014 (8)	−0.0066 (9)	0.0022 (8)
C19A	0.0341 (8)	0.0353 (8)	0.0376 (8)	0.0053 (6)	−0.0039 (6)	0.0017 (6)
C20A	0.0424 (11)	0.0441 (9)	0.0527 (12)	0.0110 (8)	−0.0112 (8)	0.0018 (9)
C21A	0.0414 (10)	0.0356 (10)	0.0431 (13)	0.0014 (8)	−0.0066 (9)	0.0022 (8)

### Geometric parameters (Å, º)

**Table:**

O1—C2	1.3609 (15)	C13—C14	1.3863 (19)
O1—HO1	0.847 (9)	C13—H13	0.9500
O2—C3	1.3690 (15)	C14—C15	1.393 (2)
O2—C7	1.4265 (15)	C14—H14	0.9500
O3—C9	1.2167 (16)	C15—C16	1.392 (2)
N1—C8	1.2847 (17)	C15—C18	1.5146 (19)
N1—N2	1.3706 (14)	C16—C17	1.381 (2)
N2—C9	1.3594 (17)	C16—H16	0.9500
N2—HN2	0.902 (9)	C17—H17	0.9500
C1—C2	1.3999 (17)	C18—C19A	1.453 (8)
C1—C6	1.4042 (18)	C18—C19	1.513 (2)
C1—C8	1.4545 (17)	C18—H18A	0.9900
C2—C3	1.4011 (17)	C18—H18B	0.9900
C3—C4	1.3802 (18)	C19—C21	1.505 (3)
C4—C5	1.397 (2)	C19—C20	1.529 (2)
C4—H4	0.9500	C19—H19	1.0000
C5—C6	1.3751 (19)	C20—H20A	0.9800
C5—H5	0.9500	C20—H20B	0.9800
C6—H6	0.9500	C20—H20C	0.9800
C7—H7A	0.9800	C21—H21A	0.9800
C7—H7B	0.9800	C21—H21B	0.9800
C7—H7C	0.9800	C21—H21C	0.9800
C8—H8	0.9500	C19A—C21A	1.50 (3)
C9—C10	1.5272 (17)	C19A—C20A	1.50 (3)
C10—C12	1.5174 (17)	C19A—H19A	1.0000
C10—C11	1.5324 (19)	C20A—H20D	0.9800
C10—H10	1.0000	C20A—H20E	0.9800
C11—H11A	0.9800	C20A—H20F	0.9800
C11—H11B	0.9800	C21A—H21D	0.9800
C11—H11C	0.9800	C21A—H21E	0.9800
C12—C17	1.3896 (18)	C21A—H21F	0.9800
C12—C13	1.3907 (18)		
			
C2—O1—HO1	106.1 (14)	C13—C14—H14	119.6
C3—O2—C7	117.83 (10)	C15—C14—H14	119.6
C8—N1—N2	118.14 (11)	C16—C15—C14	117.78 (12)
C9—N2—N1	118.74 (10)	C16—C15—C18	119.51 (14)
C9—N2—HN2	121.8 (11)	C14—C15—C18	122.71 (14)
N1—N2—HN2	118.3 (11)	C17—C16—C15	121.48 (13)
C2—C1—C6	118.76 (12)	C17—C16—H16	119.3
C2—C1—C8	121.27 (11)	C15—C16—H16	119.3
C6—C1—C8	119.97 (11)	C16—C17—C12	120.70 (13)
O1—C2—C1	123.27 (11)	C16—C17—H17	119.6
O1—C2—C3	116.91 (11)	C12—C17—H17	119.6
C1—C2—C3	119.82 (11)	C19A—C18—C15	128.4 (7)
O2—C3—C4	125.61 (11)	C19—C18—C15	116.46 (13)
O2—C3—C2	113.71 (11)	C19—C18—H18A	108.2
C4—C3—C2	120.68 (12)	C15—C18—H18A	108.2
C3—C4—C5	119.52 (12)	C19—C18—H18B	108.2
C3—C4—H4	120.2	C15—C18—H18B	108.2
C5—C4—H4	120.2	H18A—C18—H18B	107.3
C6—C5—C4	120.36 (12)	C21—C19—C18	113.35 (14)
C6—C5—H5	119.8	C21—C19—C20	110.26 (15)
C4—C5—H5	119.8	C18—C19—C20	109.95 (14)
C5—C6—C1	120.86 (12)	C21—C19—H19	107.7
C5—C6—H6	119.6	C18—C19—H19	107.7
C1—C6—H6	119.6	C20—C19—H19	107.7
O2—C7—H7A	109.5	C19—C20—H20A	109.5
O2—C7—H7B	109.5	C19—C20—H20B	109.5
H7A—C7—H7B	109.5	H20A—C20—H20B	109.5
O2—C7—H7C	109.5	C19—C20—H20C	109.5
H7A—C7—H7C	109.5	H20A—C20—H20C	109.5
H7B—C7—H7C	109.5	H20B—C20—H20C	109.5
N1—C8—C1	119.85 (11)	C19—C21—H21A	109.5
N1—C8—H8	120.1	C19—C21—H21B	109.5
C1—C8—H8	120.1	H21A—C21—H21B	109.5
O3—C9—N2	123.24 (12)	C19—C21—H21C	109.5
O3—C9—C10	123.52 (12)	H21A—C21—H21C	109.5
N2—C9—C10	113.23 (11)	H21B—C21—H21C	109.5
C12—C10—C9	109.42 (10)	C18—C19A—C21A	111.2 (18)
C12—C10—C11	113.75 (11)	C18—C19A—C20A	112.3 (16)
C9—C10—C11	108.38 (11)	C21A—C19A—C20A	132 (2)
C12—C10—H10	108.4	C18—C19A—H19A	96.7
C9—C10—H10	108.4	C21A—C19A—H19A	96.7
C11—C10—H10	108.4	C20A—C19A—H19A	96.7
C10—C11—H11A	109.5	C19A—C20A—H20D	109.5
C10—C11—H11B	109.5	C19A—C20A—H20E	109.5
H11A—C11—H11B	109.5	H20D—C20A—H20E	109.5
C10—C11—H11C	109.5	C19A—C20A—H20F	109.5
H11A—C11—H11C	109.5	H20D—C20A—H20F	109.5
H11B—C11—H11C	109.5	H20E—C20A—H20F	109.5
C17—C12—C13	118.13 (12)	C19A—C21A—H21D	109.5
C17—C12—C10	122.10 (11)	C19A—C21A—H21E	109.5
C13—C12—C10	119.77 (11)	H21D—C21A—H21E	109.5
C14—C13—C12	121.14 (12)	C19A—C21A—H21F	109.5
C14—C13—H13	119.4	H21D—C21A—H21F	109.5
C12—C13—H13	119.4	H21E—C21A—H21F	109.5
C13—C14—C15	120.75 (13)		
			
C8—N1—N2—C9	179.02 (11)	O3—C9—C10—C11	−58.49 (16)
C6—C1—C2—O1	−179.68 (12)	N2—C9—C10—C11	121.52 (12)
C8—C1—C2—O1	0.51 (19)	C9—C10—C12—C17	−73.14 (15)
C6—C1—C2—C3	0.03 (18)	C11—C10—C12—C17	48.22 (17)
C8—C1—C2—C3	−179.78 (11)	C9—C10—C12—C13	106.89 (13)
C7—O2—C3—C4	2.85 (19)	C11—C10—C12—C13	−131.75 (13)
C7—O2—C3—C2	−176.72 (11)	C17—C12—C13—C14	−0.01 (19)
O1—C2—C3—O2	−0.43 (16)	C10—C12—C13—C14	179.96 (11)
C1—C2—C3—O2	179.84 (11)	C12—C13—C14—C15	−0.6 (2)
O1—C2—C3—C4	179.97 (11)	C13—C14—C15—C16	0.3 (2)
C1—C2—C3—C4	0.25 (18)	C13—C14—C15—C18	179.73 (13)
O2—C3—C4—C5	−179.74 (13)	C14—C15—C16—C17	0.7 (2)
C2—C3—C4—C5	−0.20 (19)	C18—C15—C16—C17	−178.79 (14)
C3—C4—C5—C6	−0.1 (2)	C15—C16—C17—C12	−1.3 (2)
C4—C5—C6—C1	0.4 (2)	C13—C12—C17—C16	1.0 (2)
C2—C1—C6—C5	−0.4 (2)	C10—C12—C17—C16	−179.00 (13)
C8—C1—C6—C5	179.46 (13)	C16—C15—C18—C19A	−96.1 (12)
N2—N1—C8—C1	−178.45 (11)	C14—C15—C18—C19A	84.4 (13)
C2—C1—C8—N1	−0.19 (18)	C16—C15—C18—C19	−139.06 (16)
C6—C1—C8—N1	180.00 (12)	C14—C15—C18—C19	41.5 (2)
N1—N2—C9—O3	6.17 (18)	C15—C18—C19—C21	56.4 (2)
N1—N2—C9—C10	−173.84 (10)	C15—C18—C19—C20	−179.71 (16)
O3—C9—C10—C12	66.06 (16)	C15—C18—C19A—C21A	−11 (3)
N2—C9—C10—C12	−113.93 (12)	C15—C18—C19A—C20A	−170.6 (16)

### Hydrogen-bond geometry (Å, º)

**Table:**

*D*—H···*A*	*D*—H	H···*A*	*D*···*A*	*D*—H···*A*
O1—H*O*1···N1	0.85 (1)	1.83 (1)	2.5914 (13)	149 (2)
N2—H*N*2···O1^i^	0.90 (1)	2.40 (1)	3.2470 (14)	157 (1)
N2—H*N*2···O2^i^	0.90 (1)	2.18 (1)	2.8745 (14)	133 (1)
C10—H10···O1^i^	1.00	2.63	3.5545 (16)	155

Symmetry code: (i) *x*, −*y*+3/2, *z*−1/2.
